# Clinical Efficacy of Endoscopic Marsupialization in the Management of a Symptomatic Simple Renal Cyst: A Case Report and Review of the Current Literature

**DOI:** 10.7759/cureus.64199

**Published:** 2024-07-09

**Authors:** Saeed Bin Hamri, Abdulaziz Alhussaini, Fahad Barayan, Omar Alfraidi, Faisal Balaraj, Yasser A Noureldin

**Affiliations:** 1 Urology, Surgery, Ministry of National Guard - Health Affairs, Riyadh, SAU; 2 Urology, King Saud Bin Abdulaziz University for Health Sciences, Riyadh, SAU; 3 Urology, King Abdulaziz Medical City, Riyadh, SAU; 4 Urology, Northern Ontario School of Medicine University, Ontario, CAN

**Keywords:** renal cyst, flexible ureteroscope, holmium laser, endoscopic marsupialization, simple renal cyst

## Abstract

Simple renal cysts are commonly acquired benign lesions of the kidney. Requiring management only when it causes pain, obstruction, or gross hematuria, endoscopic marsupialization of simple renal cysts is a new method for the management of renal cysts. Herein, we present a rare case of a 44-year-old female with a simple renal cyst that was managed for the first time in Saudi Arabia by endoscopic marsupialization and discuss its efficacy and outcome compared to other methods of management.

## Introduction

Renal cysts are commonly acquired benign lesions of the kidney. They are classified according to the imaging characteristics into simple and complex renal cysts, where simple renal cysts are more common in normal kidneys [1]. It affects almost 50% of people above the age of 50 years old, with the majority are asymptomatic. Advancing age was found as a common risk factor, but the exact cause is still not well defined [2]. Management of simple renal cysts includes cyst aspiration, surgical resection, and sclerotherapy [3]. Although cyst aspiration and sclerotherapy have with higher incidence of microscopic hematuria, low-grade fever, and multiple treatments to fully ablate the kidney cyst, no intervention appears to have a better outcome than the others. Endoscopic marsupialization of simple renal cysts is a new method for the management of renal cysts [4]. Herein, we present a rare case of a simple renal cyst that was managed for the first time in Saudi Arabia by endoscopic marsupialization discussing its efficacy and outcome compared to other methods of management.

## Case presentation

A 44-year-old female, medically free with a history of cesarean section and appendectomy, presented to the hospital with a history of severe on and off left flank pain associated with nausea and fever for almost a year. These symptoms were increasing in severity over the last three months, which required several emergency room (ER) visits with the need for medications. She had no history of vomiting, hematuria, or stone formation. Physical examination revealed a healthy woman, with a vitally stable, soft, and lax abdomen with a Pfannenstiel scar. Her laboratories were within normal, including creatinine level, white blood cell level, and blood urea nitrogen (BUN). The last urine culture one week before the procedure revealed an infection with Staphylococcus coagulase negative. The patient was started empirically on ceftriaxone. An abdomen and pelvic CT urogram scan was performed to rule out ureteric stricture and revealed multiple left para-pelvic cysts, the largest measuring 4 cm without the presence of ureteric stricture. The patient underwent uneventful endoscopic marsupialization of a simple renal cyst with left double-J stent insertion.

In the operation room, a flexible ureteroscope (OTU Inc., Union City, CA) was introduced through the ureteric access sheath (12/14 FR; Rocamed Inc., Minneapolis, MN) inside the kidney, and systematic pyeloscopy was done. Two simple renal cysts were found bulging into the upper pole and middle pole of the kidney.

**Figure 1:**
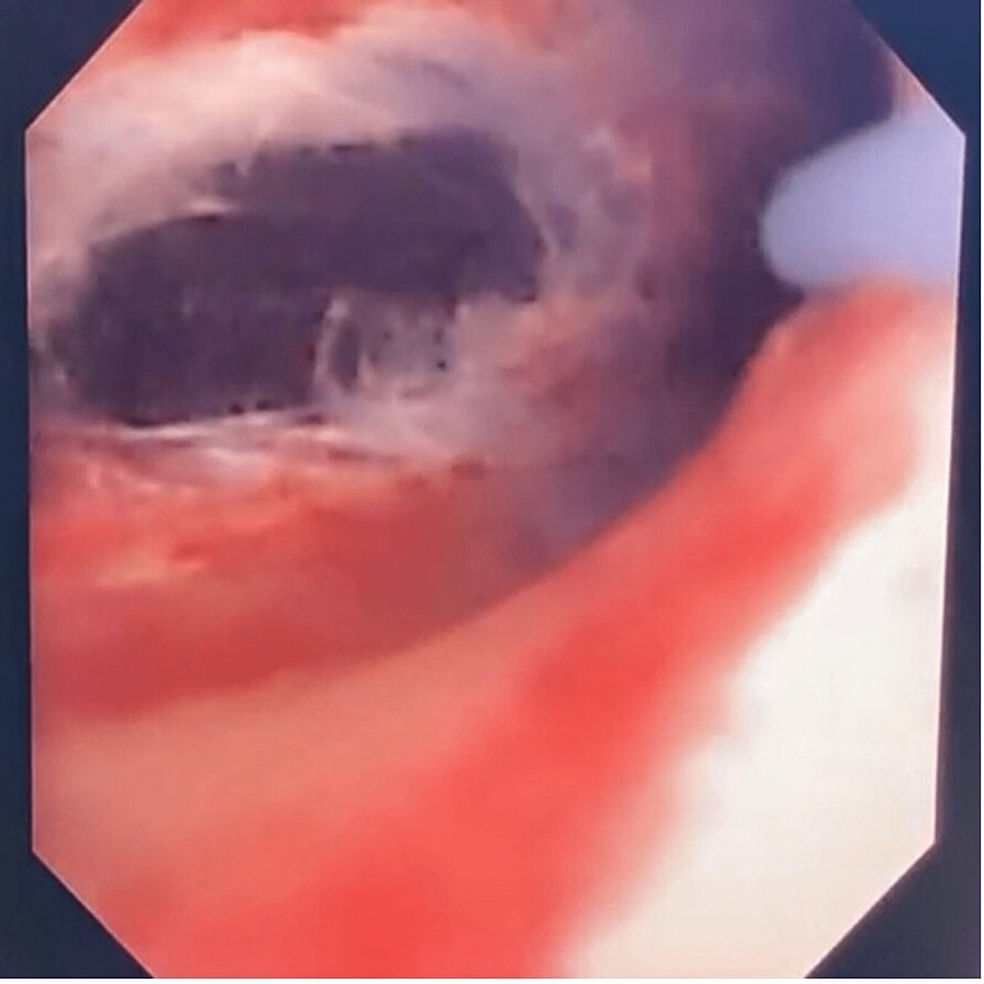
Intra-operative picture showing laser incision of the para-pelvic cyst extending into the middle pole.

**Figure 2:**
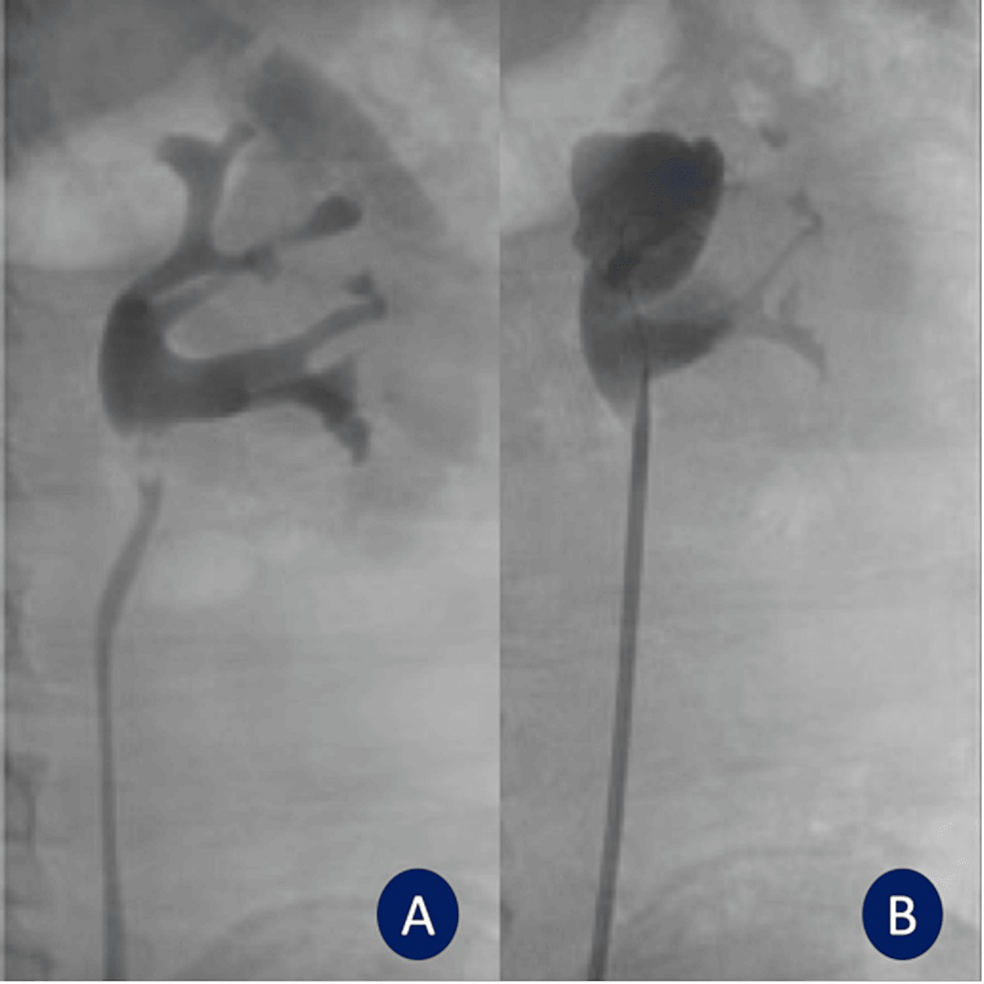
Fluoroscopy imaging: (A) pre-laser incision and (B) post-laser incision.

By using a holmium laser (0.6 energy/20 frequency; Lumenis 200, UK), an endoscopic incision of the upper pole cyst was made. The scope was advanced into the cyst to inspect for suspicious lesions or stones. Then, endoscopic marsupialization was done to the second cyst, which was in the middle pole (Figure 1). Additionally, the scope was advanced inside the cyst, and there was no suspicious lesion or stone. After that, under direct vision by the ureteroscope, a guidewire was placed into the upper pole through the ureteroscope, and the ureteroscope along with the ureteric access sheath was removed. Under fluoroscopic guidance, a DJ stent was inserted (size 24, French-6; BARD Inc., UK), and it was coiling nicely in the kidney and down in the bladder (Figure 2). The operation time was 60 minutes; the operation was uneventful, without intraoperative complications; and the patient was transferred to the anesthesia care unit for two hours and then discharged home on the same day.

**Figure 3:**
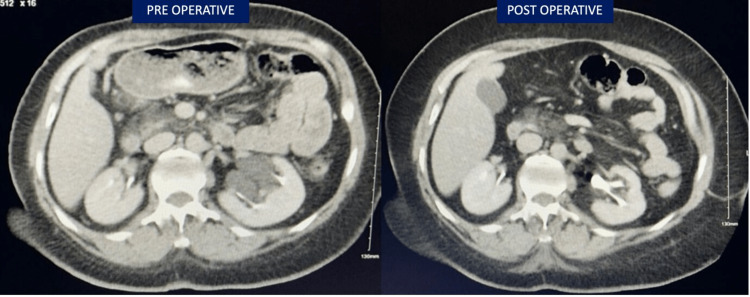
Follow-up CT scan after one year, compared with the pre-operative imaging.

She was followed up after one month in the outpatient clinic and was symptomatically improved with an unremarkable ultrasound. An abdomen and pelvic CT was done after one year post-operatively and showed resolution of para-pelvic cysts (Figure 3).

## Discussion

Simple renal cysts are sacs of fluids forming in the kidney commonly affecting older people. It rarely requires treatment. However, when it causes pain, obstruction, or gross hematuria, a surgical intervention should be considered. The simple renal cyst has multiple management methods, including percutaneous aspiration or percutaneous and laparoscopic unroofing [4,5], which has a high success rate and morbidity rate. An alternative is open cyst decortication, but it is associated with longer hospital stays alongside a high morbidity rate. Management of para-pelvic cysts poses a higher level of difficulty due to proximity to renal hilar structures. Aspiration is associated with high recurrence and obstruction rates.

Endoscopic marsupialization of simple peripheral renal cysts was recently offered as a novel method of intervention using a flexible ureteroscope (FURS) with a holmium laser [4]. To the best of our knowledge, few studies [4-6] have explored the efficacy and success of this method.

One study from a Russian group reported the outcomes of transurethral intrarenal marsupialization in nine patients with parapelvic renal cysts. The cysts were incised using FURS and holmium or thulium fiber laser and an internal stent was left inside the cyst for four to six weeks, with an average operative time of 26±11 minutes [4]. Successful outcome was reported for all patients with improvement of symptoms and decreased cavity size in follow-up CT scans [4].

Another study reported the success of 27/28 endoscopic marsupialization of para-pelvic renal cysts, without massive hemorrhage or damage to nearby organs, and the cyst size was either reduced or the cyst disappeared during an average follow-up of 39 months [5]. A large study by Meng et al. showed the success of this procedure for 31 patients with para-pelvic cysts with an average size of 5 cm, an average operative time of 30 min, and only one case of significant hemorrhage [6]. In a recent study by Huang et al., the technique was considered successful in 44/47 cases, without serious complications, except for the recurrence in three cases [7]. The investigators highlighted the potentially vital role of the intraoperative real-time ultrasound in the identification of the cyst during this technique [7]. Our case is within line with the findings of published literature in terms of the short operative time, potentially excellent success, and minimal morbidity, as there was no significant hemorrhage or nearby organ injury and the success was confirmed both subjectively, by improved patient symptoms, and objectively, by CT urogram.

## Conclusions

Simple renal cysts are very common and mostly asymptomatic. However, when it becomes symptomatic, intervention is required. Endoscopic marsupialization is a quite new method for managing simple renal cysts.
